# Increased risk of hyperthyroidism induced by immune checkpoint inhibitors in patients with existing or prior Graves’ disease: a nested prospective cohort study with propensity score matching

**DOI:** 10.3389/fendo.2025.1701500

**Published:** 2025-12-01

**Authors:** Koji Suzuki, Tomoko Kobayashi, Tetsushi Izuchi, Takanori Murase, Masahiko Ando, Tomoko Handa, Takeshi Onoue, Takashi Miyata, Mariko Sugiyama, Daisuke Hagiwara, Hidetaka Suga, Ryoichi Banno, Hiroshi Arima, Shintaro Iwama

**Affiliations:** 1 Department of Endocrinology and Diabetes, Nagoya University Graduate School of Medicine, Nagoya, Japan; 2 Center for Advanced Medicine and Clinical Research, Nagoya University Hospital, Nagoya, Japan; 3 Department of Clinical Research Education, Nagoya University Graduate School of Medicine, Nagoya, Japan; 4 Research Center of Health, Physical Fitness and Sports, Nagoya University, Nagoya, Japan

**Keywords:** hyperthyroidism, PD-1 inhibitor, CTLA-4 inhibitor, Graves’, recurrence

## Abstract

**Background:**

Thyroid dysfunction induced by immune checkpoint inhibitors (ICIs) commonly manifests as destructive thyroiditis and hypothyroidism, while hyperthyroidism (Graves’ disease) is rare. However, the clinical characteristics of thyroid dysfunction in patients with existing or prior Graves’ disease treated with ICIs remain unclear.

**Methods:**

A case-control study was performed using a prospective cohort of patients treated with ICIs between November 2015 and January 2024. At baseline, cases had Graves’ disease without overt thyroid dysfunction or prior Graves’ disease and were matched 1:5 with controls by age, gender, antithyroid antibody status at baseline, and ICI type. The incidence of thyroid dysfunction induced by ICIs was compared between cases and controls. The incidence of hyperthyroidism was compared with the exacerbation rate in ICI-untreated outpatients with Graves’ disease who were in remission or had no overt thyroid dysfunction for ≥1 year without changes in low-dose antithyroid medication.

**Results:**

Nineteen patients (13 in remission and 6 receiving thiamazole at baseline) were included and matched with 95 controls. During follow-up, 5 cases negative for thyroid-stimulating hormone receptor antibody (TRAb) at baseline developed thyrotoxicosis: 3 were TRAb-positive (2 with increased uptake on thyroid scintigraphy) suggesting hyperthyroidism, and 2 had destructive thyroiditis (TRAb-negative). The incidence of hyperthyroidism was significantly higher in cases than in controls (3/19 [15.8%] vs 0/95 [0%], *p* < 0.05), whereas the incidence of destructive thyroiditis (2/19 [10.5%] vs. 15/95 [15.8%], *p* = 0.734) or isolated hypothyroidism (0/19 [0%] vs. 13/95 [13.7%], *p* = 0.121) did not differ between groups. The incidence of hyperthyroidism was also higher in cases than in outpatients with ICI-unrelated Graves’ disease (3/19 [15.8%] vs 10/269 [3.7%], respectively; *p* < 0.05).

**Conclusion:**

Patients with existing or prior Graves’ disease have an increased risk of hyperthyroidism following ICI treatment, highlighting their need for careful differential diagnosis of thyrotoxicosis.

## Introduction

Immune checkpoint inhibitors (ICIs), which block immune checkpoints to enable T cells to attack cancer cells, are widely used in the treatment of advanced malignancies. However, ICIs can also cause immune-related adverse events (irAEs), which commonly affect the lung, skin, gastrointestinal tract, liver, and endocrine glands (1–3). Thyroid dysfunction is one of the most frequently observed endocrine irAEs (4–8), and consists of thyrotoxicosis and hypothyroidism. While destructive thyroiditis resulting from immune-mediated thyroid tissue damage (9) is a common cause of thyrotoxicosis, hyperthyroidism following ICI therapy is rare, and its underlying mechanisms and risk factors remain unclear (10).

In recent years, several studies have reported the risk of exacerbation of pre-existing autoimmune diseases following ICI therapy. A meta-analysis of observational studies reported that 219 of 619 patients (35%, 95% confidence interval [CI]: 29–41%) experienced exacerbation of their autoimmune disease condition, including 40 of 95 (42%) patients with pre-existing autoimmune thyroiditis who experienced exacerbation (11). On the other hand, reports on the exacerbation of Graves’ disease in patients treated with ICI are limited. In a previous retrospective study, one out of eight (12.5%) patients with Graves’ disease was reported to have experienced an exacerbation (12). However, the frequency of hormone measurements to assess an exacerbation, as well as the details of diagnostic evaluations performed at the time of the exacerbation, were not clearly described. Furthermore, no prospective studies have investigated whether patients with existing or prior Graves’ disease are more likely to experience disease exacerbation following ICI therapy. In addition, it remains unclear whether these patients have a higher incidence of thyroid irAEs other than hyperthyroidism following treatment with ICIs.

We conducted a matched case-control study using data from a prospective cohort to clarify the clinical characteristics of ICI-induced thyroid dysfunction in patients with existing or prior Graves’ disease.

## Methods

### Patient selection

This prospective study enrolled all patients with existing or prior Graves’ disease who initiated nivolumab, pembrolizumab, atezolizumab, durvalumab, or ipilimumab combined with nivolumab therapy at Nagoya University Hospital between November 2, 2015 and January 31, 2024 (UMIN000019024). To compare the incidence of thyroid irAEs between patients with existing or prior Graves’ disease (GD group) and those without, the Control 1 cohort was established, which comprised patients without a history of Graves’ disease who began the same ICI therapy during the same period. Exclusion criteria were those with prior ICI therapy, combination therapy with tyrosine kinase inhibitors (TKIs), history of total or subtotal thyroidectomy, history of radioiodine therapy, and a thyroid-function follow-up of less than 12 weeks in the GD group or less than 48 weeks in the Control 1 group. Follow-up was censored at death, loss to thyroid-function monitoring, a switch to TKIs or other ICIs, or at the end of observation on January 31, 2025. The study was approved by the Ethics Committee of Nagoya University Hospital (Approval No. 2015-0273), conducted in accordance with the Declaration of Helsinki, and all participants provided written informed consent.

The second control group (Control 2) was established retrospectively to compare the incidence of hyperthyroidism in the GD group with the one-year exacerbation rate in outpatients with Graves’ disease without ICI therapy. Outpatients with a registered diagnosis of “Graves’ disease” or “hyperthyroidism” at Nagoya University Hospital between April 1, 2017 and March 26, 2025 were screened. Exclusion criteria were unconfirmed diagnostic criteria of the Japan Thyroid Association (13) upon review of the medical records, history of ICI therapy, thyroidectomy or radioiodine treatment, or lack of thyroid function follow-up for at least two years prior to the most recent evaluation. To ensure comparability, outpatients with Graves’ disease who were in remission or receiving low-dose thiamazole (≤10 mg/day) were selected as Control 2 group members, matching the clinical status of the ICI-treated group. The two-year follow-up period was divided into two phases. The first year was a stability confirmation period; patients who developed thyrotoxicosis or required changes in antithyroid medication during this phase were excluded. The second year served as the observation period, during which the exacerbation rate of Graves’ disease was assessed. The analysis using patients in the Control 2 group was conducted with institutional approval, following disclosure in accordance with institutional ethical guidelines.

### Data collection

In both the GD and Control 1 groups, serum levels of free triiodothyronine (FT3), free thyroxine (FT4), and thyroid-stimulating hormone (TSH) were measured at baseline and 6, 12, 18, 24, 36, and 48 weeks after the first administration of ICIs, with additional measurements taken as clinically indicated. Levels of thyroglobulin antibody (TgAb) and thyroid peroxidase antibody (TPOAb) were measured at baseline in all patients. Serum levels of TRAb were measured at baseline in the GD group and at thyrotoxicosis onset in all patients. In the Control 2 group, thyroid function tests were administered based on the clinical judgment of the attending physicians. If the baseline TRAb level was unavailable, the most recent measurement obtained prior to the observation period was used as a substitute. Serum levels of FT3, FT4 and TSH were measured using chemiluminescence immunoassay (Abbott Diagnostics, Santa Clara, CA), and normal reference ranges were: FT3 1.71–3.71 pg/mL, FT4 0.70–1.48 ng/dL, and TSH 0.35–4.94 μIU/mL, respectively. TPOAb, TgAb, and TRAb were measured using electrochemiluminescence assays (Roche Diagnostics, Mannheim, Germany), with normal ranges of <16 IU/mL, <28 IU/mL, and <2.0 IU/mL, respectively. When the baseline TRAb level was below the assay’s detection limit, a titer equal to the lower limit of detection was assigned.

### Definition of thyroid dysfunction

Thyroid irAEs were defined based on Japan Endocrine Society guidelines (6) and categorized as thyrotoxicosis or isolated hypothyroidism. Thyrotoxicosis was further subclassified into hyperthyroidism (TRAb-positive) and destructive thyroiditis (TRAb-negative). In accordance with the guidelines for the diagnosis of Graves’ disease from the Japan Thyroid Association (13), cases presenting with thyrotoxicosis and positive for TRAb were diagnosed as probable hyperthyroidism, while those also showing increased uptake on thyroid scintigraphy were diagnosed as definitive hyperthyroidism.

In patients with existing or prior Graves’ disease, the development of hyperthyroidism after remission was defined as a relapse, while the development of hyperthyroidism during antithyroid treatment was defined as a flare. In the Control 2 group, TRAb levels were not measured at the onset of thyrotoxicosis in some cases. In such instances, an exacerbation of Graves’ disease was determined based on the attending physician’s clinical judgment, as indicated by the resumption or escalation of antithyroid medication.

### Propensity score matching

We used logistic regression to estimate propensity scores, including age, gender, baseline antithyroid antibody status, and type of ICI. Patients in the GD group were matched in a 1:5 ratio with those in the Control 1 group using nearest-neighbor matching without replacement. The initial caliper width was set at 0.2 standard deviations of the logit of the propensity score, as commonly recommended. However, to address the exclusion of one patient from the GD group and to accommodate the overall small sample size, the caliper width was increased to 0.3 standard deviations to minimize further patient loss. Covariate balance between the matched groups was assessed post-matching.

### Statistical analysis

All statistical analyses were conducted using EZR (Jichi Medical University, Tochigi, Japan). Continuous variables were tested for normality via Shapiro–Wilk. Normally distributed data are presented as mean ± standard deviation, and non-normal as median (interquartile range). Categorical variables are expressed as frequencies and percentages, and 95% CIs are provided for group comparisons. Group comparisons used t-tests or Mann–Whitney U tests for continuous data and chi-square or Fisher’s exact tests for categorical variables. A logistic regression model was performed to identify potential explanatory factors for the incidence of hyperthyroidism. Two-tailed *p*-values <0.05 were considered significant.

## Results

### Baseline characteristics

**Figure 1:**
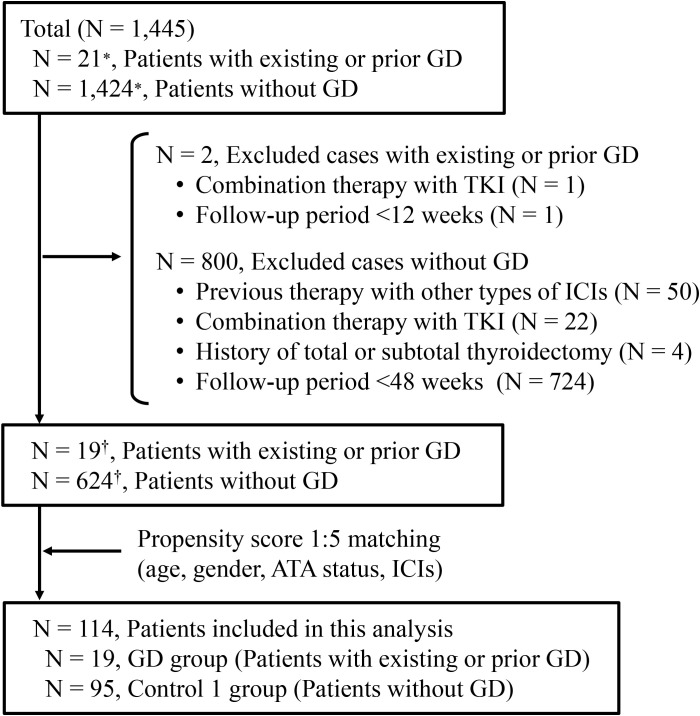
Enrollment of patients treated with immune checkpoint inhibitors. ^*^Reflects registration entries rather than individual cases, as some patients were enrolled multiple times for different treatments in our clinical study. ^†^Reflects the number of unique patients after removing duplicates. GD, Graves’ disease; TKI, tyrosine kinase inhibitor; ATA, antithyroid antibody; ICI, immune checkpoint inhibitor.

Among the 1,445 registered cases included in the prospective cohort during the study period, 802 cases were excluded according to the exclusion criteria, as shown in **Figure 1**. As a result, 19 patients with existing or prior Graves’ disease and 624 patients without a history of Graves’ disease were identified (**Figure 1**). The baseline clinical characteristics of the GD group are shown in **Table 1**. In the GD group, the median age at ICI initiation was 69 years (60–70), and 52.6% of the patients were male. Lung cancer was the most common malignancy (n = 8, 42.1%). At baseline, 13 patients were in remission of Graves’ disease, and six were receiving thiamazole; none exhibited overt thyroid dysfunction. One patient was TRAb-positive. The ICI regimens included nivolumab (n = 5), pembrolizumab (n = 8), atezolizumab (n = 4), durvalumab (n = 1), and a combination of ipilimumab and nivolumab (n = 1). After propensity score matching, 19 patients with existing or prior GD were matched with 95 controls (Control 1), as shown in **Figure 1**. The clinical characteristics of the two groups demonstrated balance in age, gender, antithyroid antibody status at baseline and ICI therapy, which retained a standardized difference of <0.20 (**Table 2**). The median duration of thyroid function follow-up was significantly longer in the matched control group than in the GD group [490 (351–699) vs 308 (136–495) days, respectively; *p* = 0.001] (**Table 2**).

**Table 1:** Baseline clinical characteristics of patients with existing or prior Graves’ disease treated with immune checkpoint inhibitors.

	GD group, n = 19
	No. (%) or Median (IQR)
Age (range), years	69 (60–70)
Gender	
Female	9 (47.4%)
Male	10 (52.6%)
Cancer type	
Lung	8 (42.1%)
Bladder	2 (10.5%)
Cervix	2 (10.5%)
Head and neck	2 (10.5%)
Biliary tract	1 (5.3%)
Breast	1 (5.3%)
Kidney	1 (5.3%)
Liver	1 (5.3%)
Stomach	1 (5.3%)
GD status at baseline	
In remission	13 (68.4%)
Receiving MMI treatment	6 (31.6%)
TF at baseline	
Euthyroid	16 (84.2%)
Subclinical hyperthyroidism	3 (15.8%)
Overt thyroid dysfunction	0 (0%)
TRAb at baseline	
Positive	1 (5.3%)
Negative	18 (94.7%)
TgAb at baseline	
Positive	7 (36.8%)
Negative	12 (63.2%)
TPOAb at baseline	
Positive	7 (36.8%)
Negative	12 (63.2%)
ICI therapy	
Nivolumab	5 (26.3%)
Pembrolizumab	8 (42.1%)
Atezolizumab	4 (21.1%)
Durvalumab	1 (5.3%)
Ipilimumab/Nivolumab	1 (5.3%)

GD, Graves’ disease; IQR, interquartile range; MMI, thiamazole; TF, thyroid function; TRAb, thyroid-stimulating hormone receptor antibody; TgAb, thyroglobulin antibody; TPOAb, thyroid peroxidase antibody; ICI, immune checkpoint inhibitor.

**Table 2:** Baseline characteristics of patients treated with immune checkpoint inhibitors with existing or prior Graves’ disease and without Graves’ disease after propensity score matching.

Characteristics	GD group, n = 19	Control 1, n = 95	*p* value	SD
	No. (%, 95% CI) or median (IQR)			
Age (range), years	69 (60–70)	69 (64–71)	0.547	0.172
Gender				
Female	9 (47.4%, 24.4–71.1)	43 (45.3%, 35.0–55.8)	1.000	0.042
Male	10 (52.6%, 28.9–75.6)	52 (54.7%, 44.2–65.0)		
ATA status at baseline				
Positive*	11 (57.9%, 33.5–79.7)	55 (57.9%, 47.3–68.0)	1.000	<0.001
Negative	8 (42.1%, 20.3–66.5)	40 (42.1%, 32.0–52.7)		
ICI therapy				
PD-1	13 (68.4%, 43.4–87.4)	66 (69.5%, 59.2–78.5)	0.800	0.106
PD-L1	5 (26.3%, 9.1–51.2)	26 (27.4%, 18.7–37.5)		
PD-1/CTLA4	1 (5.3%, 0.1–26.0)	3 (3.2%, 0.7–9.0)		
TF follow-up, days	308 (136–495)	490 (351–699)	0.001	0.462

*Baseline TgAb and/or TPOAb positive

GD, Graves’ disease; IQR, interquartile range; SD, standardized differences; CI, confidence interval; ATA, antithyroid antibody; ICI, immune checkpoint inhibitor; PD-1, anti-programmed cell death-1; PD-L1, anti-programmed cell death-1 ligand 1; CTLA-4, anti-cytotoxic T-lymphocyte antigen 4; TF, thyroid function; TgAb, thyroglobulin antibody; TPOAb, thyroid peroxidase antibody.

### Thyroid irAEs

Thyroid irAEs occurred in 5 of 19 patients (26.3%) in the GD group and in 28 of 95 patients (29.5%) in the matched control group (**Table 3**). In the GD group, all five affected patients who were TRAb-negative at initiation of ICI therapy presented with thyrotoxicosis at the onset. Among them, three patients became TRAb-positive at the onset of thyrotoxicosis and were diagnosed with probable hyperthyroidism; in two cases, the diagnosis was confirmed by the findings of ^99m^Tc-pertechnetate thyroid scintigraphy. The remaining two patients in the GD group developed TRAb-negative thyrotoxicosis that resolved without intervention—one to euthyroidism and the other to hypothyroidism—consistent with a clinical course of destructive thyroiditis. In the matched control group, 15 patients presented with TRAb-negative thyrotoxicosis, all of which progressed without treatment to either euthyroidism (n = 3) or hypothyroidism (n = 12), consistent with destructive thyroiditis. An additional 13 patients developed isolated hypothyroidism without a preceding thyrotoxic phase. The incidence of hyperthyroidism was significantly higher in the GD group compared with the matched control group (3/19 [15.8%] vs. 0/95 [0%], respectively; *p* = 0.004) (**Table 3**). Even when limited to definitive cases confirmed by thyroid scintigraphy, the difference remained statistically significant [2/19 (10.5%) vs. 0/95 (0%), respectively; *p* = 0.027]. In contrast, there were no significant differences between the two groups in the incidence of destructive thyroiditis [2/19 (10.5%) vs. 15/95 (15.8%), respectively; *p* = 0.734] or isolated hypothyroidism [0/19 (0%) vs. 13/95 (13.7%), respectively; *p* = 0.121].

**Table 3:** Thyroid irAEs observed following immune checkpoint inhibitor therapy.

	GD group, n = 19	Control 1, n = 95	*p* value
	No. (%, 95% CI)		
Overall thyroid irAEs	5 (26.3%, 9.1–51.2)	28 (29.5%, 20.6–39.7)	1.000
Hyperthyroidism	3 (15.8%, 3.4–39.6)	0 (0%, 0–3.8)	0.004
Destructive thyroiditis	2 (10.5%, 1.3–33.1)	15 (15.8%, 9.1–24.7)	0.734
Isolated hypothyroidism	0 (0%, 0–17.6)	13 (13.7%, 7.5–22.3)	0.121

GD, Graves’ disease; CI, confidence interval; irAE, immune-related adverse event.

### Comparison of the incidence of hyperthyroidism in the GD group with that in outpatients who were not treated with ICIs

**Figure 2:**
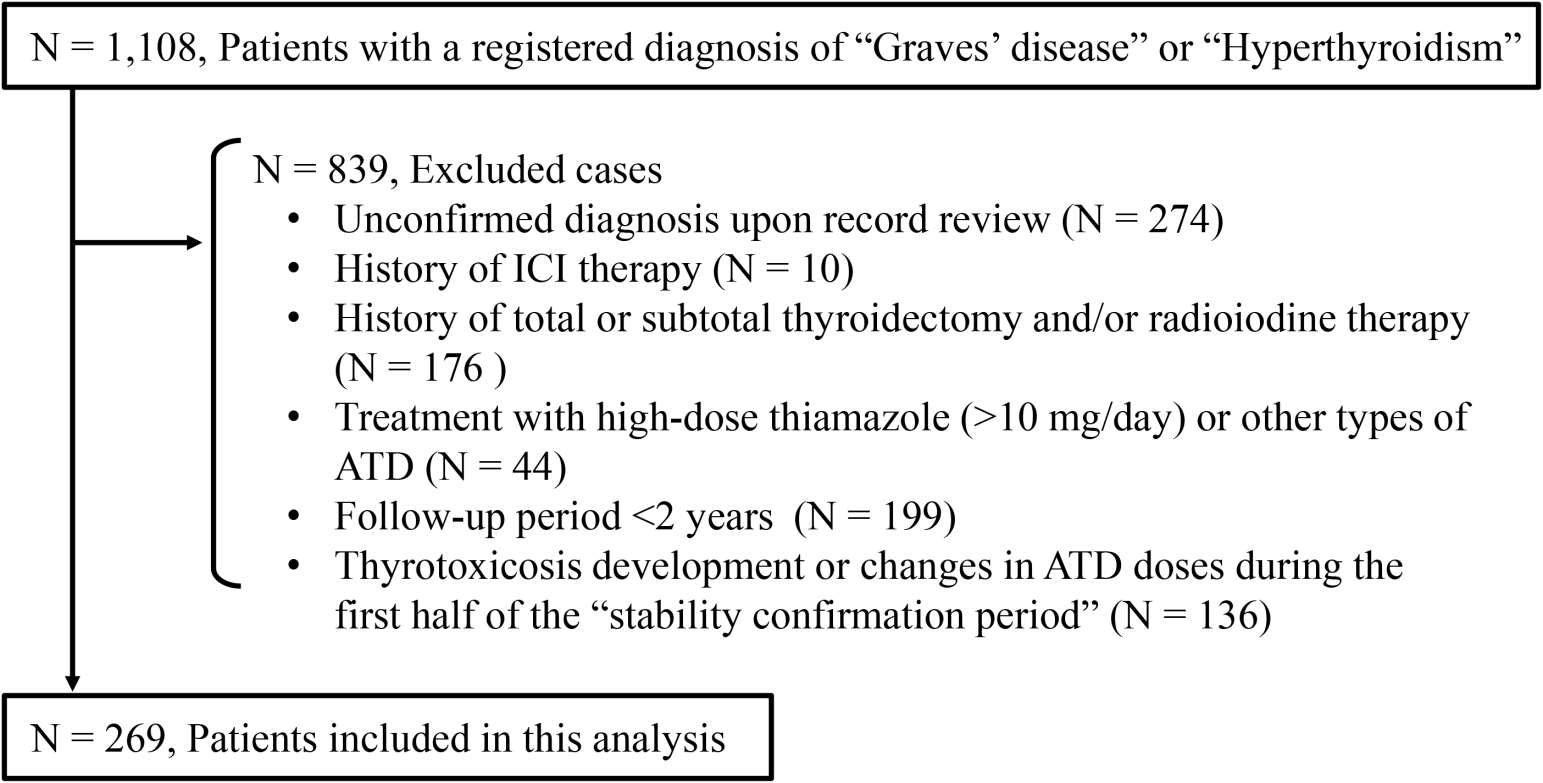
Enrollment of outpatients with Graves’ disease who had not received immune checkpoint inhibitor therapy. ICI, immune checkpoint inhibitor; ATD, antithyroid drug.

The Control 2 group comprised 269 outpatients with stable Graves’ disease (**Figure 2**). Baseline clinical characteristics of the ICI-treated GD group and the Control 2 group are shown in **Table 4**. Compared with the ICI-treated GD group, the Control 2 group included a higher proportion of females (**Table 4**), whereas no significant differences were observed in age, GD status at baseline or TRAb levels at baseline between the two groups. The incidence of hyperthyroidism in the ICI-treated GD group was significantly higher than the exacerbation rate in the Control 2 group [3/19 (15.8%) vs. 10/269 (3.7%), respectively; *p* = 0.046] (**Table 5**). Since there was a gender imbalance between the two groups, a logistic regression analysis was performed to assess the effects of gender and ICI treatment on the incidence of hyperthyroidism (**Supplementary Table S1**). In the univariate analysis, ICI treatment was significantly associated with hyperthyroidism (odds ratio 4.86, 95% CI 1.22–19.4, *p* = 0.025), and this association remained significant in the multivariate analysis, which included gender as a covariate (odds ratio 4.40, 95% CI 1.06–18.3, *p* = 0.041).

**Table 4:** Baseline clinical characteristics of patients with existing or prior Graves’ disease with or without immune checkpoint inhibitor therapy.

Characteristics	GD group, n = 19	Control 2, n = 269	*p* value
	No. (%, 95% CI) or Median (IQR)		
Age (range), years	69 (60–70)	64 (52–74)	0.453
Gender			
Female	9 (47.4%, 24.4–71.1)	202 (75.1%, 69.5–80.1)	0.014
Male	10 (52.6%, 28.9–75.6)	67 (24.9%, 19.9–30.5)	
GD status at baseline			
In remission	13 (68.4%, 43.4–87.4)	137 (50.9%, 44.8–57.1)	0.160
Receiving MMI treatment	6 (31.6%, 12.6–56.6)	132 (49.1%, 42.9–55.2)	
TRAb at baseline (IU/mL)	0.80 (0.80–0.90)	0.80 (0.80–1.40)	0.195

GD, Graves’ disease; CI, confidence interval; IQR, interquartile range; MMI, thiamazole; TRAb, thyroid-stimulating hormone receptor antibody.

**Table 5:** One-year incidence of hyperthyroidism in patients with existing or prior Graves’ disease with or without immune checkpoint inhibitor therapy.

	GD group, n = 19	Control 2, n = 269	*p* value
	No. (%, 95% CI)		
Hyperthyroidism	3 (15.8%, 3.4–39.6)	10 (3.7%, 1.8–6.7)	0.046

GD, Graves’ disease; CI, confidence interval.

### Clinical summaries of three patients in the GD group who developed hyperthyroidism during ICI therapy

#### Case 1

Case 1 was diagnosed with Graves’ disease at age 64 and achieved remission after one year of thiamazole treatment. At age 76, he began adjuvant nivolumab therapy after surgical resection of bladder cancer. At nivolumab initiation, thyroid function was normal, with negative TRAb. After the eighth cycle of nivolumab on day 151, the patient began to present with palpitations, sweating and fatigue. On day 179, laboratory tests revealed overt thyrotoxicosis (TSH: 0.0090 μIU/mL, FT3: 5.33 pg/mL, FT4: 1.86 ng/dL), along with seroconversion to TRAb positivity (3.7 IU/mL). Thyroid ultrasonography showed decreased internal echogenicity without diffuse enlargement or apparent increased vascularity. As thyrotoxicosis persisted for one month, ^99m^Tc-pertechnetate thyroid scintigraphy was performed. Although the uptake rate was 1.2%, which falls within the normal range, diffuse tracer accumulation was observed in both thyroid lobes despite TSH suppression. Based on these findings, a relapse of Graves’ disease was diagnosed, and thiamazole (10 mg/day) was initiated. While thyroid hormone levels normalized promptly, the patient remained TRAb-positive with persistently suppressed TSH, and thiamazole was continued. After 14 cycles, nivolumab was discontinued due to pulmonary toxicity. Eighteen months after the initiation of ICI therapy, the patient remains progression-free, with well-controlled Graves’ disease on thiamazole (5 mg/day).

#### Case 2

Case 2 was diagnosed with Graves’ disease at age 58, and she achieved remission by age 64 following treatment with thiamazole. Pembrolizumab was initiated at age 70 due to the recurrence of lung cancer. Baseline thyroid function was normal, and TRAb was negative. On day 166, after five cycles of pembrolizumab, routine laboratory tests revealed thyrotoxicosis (FT3: 5.24 pg/mL, FT4: 2.33 ng/mL, TSH: 0.0069 μIU/mL) with seroconversion to TRAb positivity (2.3 IU/mL). Thyroid ultrasound showed diffuse enlargement, increased parenchymal heterogeneity and mild hypervascularity. ^99m^Tc-pertechnetate scintigraphy demonstrated increased radioactive iodine uptake (7.1%), and the patient was diagnosed with a relapse of Graves’ disease. Following discontinuation of ICI therapy, thyroid hormone levels normalized spontaneously, and TRAb became negative by day 208. Subsequently, ICI therapy was resumed. After 13 cycles, pembrolizumab was discontinued due to skin toxicity. As of six years after initiation of therapy, she remains free from disease progression, with stable, euthyroid function. A part of the clinical course of this case has already been reported previously (14).

#### Case 3

Case 3 was diagnosed with Graves’ disease at age 63, and he started thiamazole therapy. Due to recurrence of lung cancer at age 69, nivolumab therapy was initiated. At the time, his Graves’ disease was stable on thiamazole (5 mg every other day), with normal baseline thyroid function and negative TRAb (1.8 IU/mL). Baseline TPOAb was positive (18.9 IU/mL), and TgAb was negative. Baseline thyroglobulin was mildly elevated at 79.1 ng/mL. On day 46, after one cycle of nivolumab, routine laboratory tests showed thyrotoxicosis (FT3: 1.97 pg/mL, FT4: 1.64 ng/dL, TSH: 0.2669 μIU/mL) with TRAb positivity (2.1 IU/mL). At the onset of thyrotoxicosis, TPOAb became negative, and TgAb remained negative. The thyroglobulin level decreased to 57.9 ng/mL. Based on these findings, the patient was diagnosed with a flare of Graves’ disease. Thyroid function normalized within six weeks without any changes to the thiamazole regimen, and TRAb status returned to negative (1.1 IU/mL). After one additional cycle of nivolumab, the patient’s lung cancer progressed, resulting in discontinuation of ICI therapy. He died of lung cancer at age 70.

## Discussion

This case-control study is, to our knowledge, the first to demonstrate that patients with existing or prior Graves’ disease have an increased risk of developing hyperthyroidism following ICI treatment compared with those without a history of Graves’ disease. Furthermore, the incidence of hyperthyroidism in these patients is higher than the exacerbation rate observed in patients with Graves’ disease that had been in remission without antithyroid medication or in a long-term stable state with low doses of antithyroid medication.

Thyroid dysfunction induced by ICIs is a common endocrine irAE, mainly manifested as destructive thyroiditis or hypothyroidism, while hyperthyroidism is rare (10, 14–20). In our previous prospective study involving 209 patients treated with anti-programmed cell death-1 (PD-1) antibodies, only one case (0.5%) was diagnosed with hyperthyroidism, based on thyroid scintigraphy (14). A recent review identified only 19 reported cases of hyperthyroidism induced by ICIs, eight of which were confirmed based on thyroid scintigraphy (21). None of the 19 patients had a history of autoimmune thyroid disease, and the risk factors for developing hyperthyroidism after ICI treatment remain unclear. The findings of this study suggest that existing or prior Graves’ disease is a risk factor for ICI-induced hyperthyroidism. In one of our cases (Case 3), thyroid scintigraphy was not performed, leaving a possibility of destructive thyroiditis as the cause of thyrotoxicosis. Nevertheless, even after excluding this case, the incidence of hyperthyroidism remained significantly higher in patients with existing or prior Graves’ disease than in those without.

Graves’ disease is known to relapse frequently after discontinuation of antithyroid medication (22–24), and three of 19 (15.8%) patients experienced an exacerbation of Graves’ disease following the initiation of ICI in the present study. However, this rate was significantly higher than that observed in a matched cohort of outpatients with Graves’ disease who were not treated with ICIs, suggesting that ICI administration increases the risk of disease exacerbation in patients with existing or prior Graves’ disease. In contrast, the incidence of thyroid irAEs other than hyperthyroidism did not differ between patients with existing or prior Grave’s disease and without Graves’ disease, suggesting that a history or current diagnosis of Graves’ disease does not confer a particularly increased risk for developing destructive thyroiditis or hypothyroidism.

The exact mechanisms by which ICIs induce hyperthyroidism remain unclear. Humoral immunity plays a critical role in the pathogenesis of Graves’ disease unrelated to ICI (25). It is known that impaired immune tolerance leads to T cell infiltration in the thyroid and activation of B cells, as well as increased synthesis and secretion of functional autoantibodies directed against the TSH receptor (26). While the use of ICIs causes activation of CD8^+^ cytotoxic T cells (cellular immunity), it may also activate autoreactive CD4^+^ T cells, which are known to express PD-1 and cytotoxic T-lymphocyte antigen 4 (CTLA-4) (27, 28), which modulate humoral immunity (29), increasing preexisting autoantibodies or inducing *de novo* development of autoantibodies. Some irAEs are thought to be mediated by humoral immunity through a functional effect of autoantibodies. For example, anti-acetylcholine receptor antibodies have been reported to increase in patients with ICI-associated myasthenia gravis (30, 31), and anti-calcium-sensing receptor antibodies to increase in patients with ICI-associated hypoparathyroidism (32). These findings suggest that ICIs may amplify an underlying predisposition to produce TRAb, resulting in the development of hyperthyroidism. Supporting this, Wang et al. reported a case of transient hyperthyroidism after atezolizumab administration followed by persistent hypothyroidism (33). Further studies are needed to clarify the pathogenesis of ICI-induced hyperthyroidism.

Our study has several limitations. First, the sample size in the GD group was relatively small. Further studies involving larger cohorts and longer observation periods will be essential to confirm the generalization and clinical relevance of our findings. Second, the safety of ICIs in patients with unstable Graves’ disease requires further investigation, as this study included only patients with Graves’ disease in which thyroid function was stable on low doses of thiamazole. Third, propensity score matching was not performed in the comparison with the outpatients with Graves’ disease unrelated to ICI (Control 2 group), resulting in a gender imbalance between groups. Moreover, the Control 2 group was retrospectively recruited, which may have served as potential confounding factors in the comparison. However, a multivariate logistic regression analysis including gender as a covariate showed the association of ICI treatment with the exacerbation of the Graves’ disease. Finally, it could not be completely ruled out that the hyperthyroidism observed in the ICI-treated GD group was due to a spontaneous exacerbation rather than an irAE. The strengths of our study are the prospective collection of data and the case-control design, which enabled a more accurate estimation of the risk of thyroid irAEs depending on the history of Graves’ disease.

In conclusion, patients with existing or prior Graves’ disease have an increased risk of developing hyperthyroidism following ICI treatment, highlighting the need for careful differential diagnosis of thyrotoxicosis in these patients.

## Data Availability

The raw data supporting the conclusions of this article will be made available by the authors upon reasonable and appropriate request.
