# Supplementary material for: Increased risk of hyperthyroidism induced by immune checkpoint inhibitors in patients with existing or prior Graves’ disease: a nested prospective cohort study with propensity score matching
Source: Front Endocrinol (Lausanne). 2025 Dec 1;16:1701500. doi: 10.3389/fendo.2025.1701500 (PMC12702723; doi:10.3389/fendo.2025.1701500)
Supplement: Supplementary file 1 [file Table1.docx]

**Supplementary Table S1. Univariate and multivariate logistic regression analyses for predicting the one-year incidence of hyperthyroidism in patients with existing or prior Graves’ disease with or without immune checkpoint inhibitor therapy**

| Variables | Univariate analysis | | | Multivariate analysis | | |
| --- | --- | --- | --- | --- | --- | --- |
|  | Odds ratio | 95% CI | *p* value | Odds ratio | 95% CI | *p* value |
| Gender |  |  |  |  |  |  |
| Female | 1 |  |  | 1 |  |  |
| Male | 1.76 | 0.55–5.56 | 0.334 | 1.44 | 0.43–4.75 | 0.552 |
| ICI treatment |  |  |  |  |  |  |
| No | 1 |  |  | 1 |  |  |
| Yes | 4.86 | 1.22–19.4 | 0.025 | 4.40 | 1.06–18.3 | 0.041 |

Abbreviations: CI, confidence interval; ICI, immune checkpoint inhibitor.
